# Supplementary material for: Genetic Basis of Potato Tuber Defects and Identification of Heat-Tolerant Clones
Source: Plants (Basel). 2024 Feb 23;13(5):616. doi: 10.3390/plants13050616 (PMC10934851; doi:10.3390/plants13050616)
Supplement: Supplementary file 1 [file plants-13-00616-s001.zip › Supplementary Figures.pdf]

Supplementary Figures

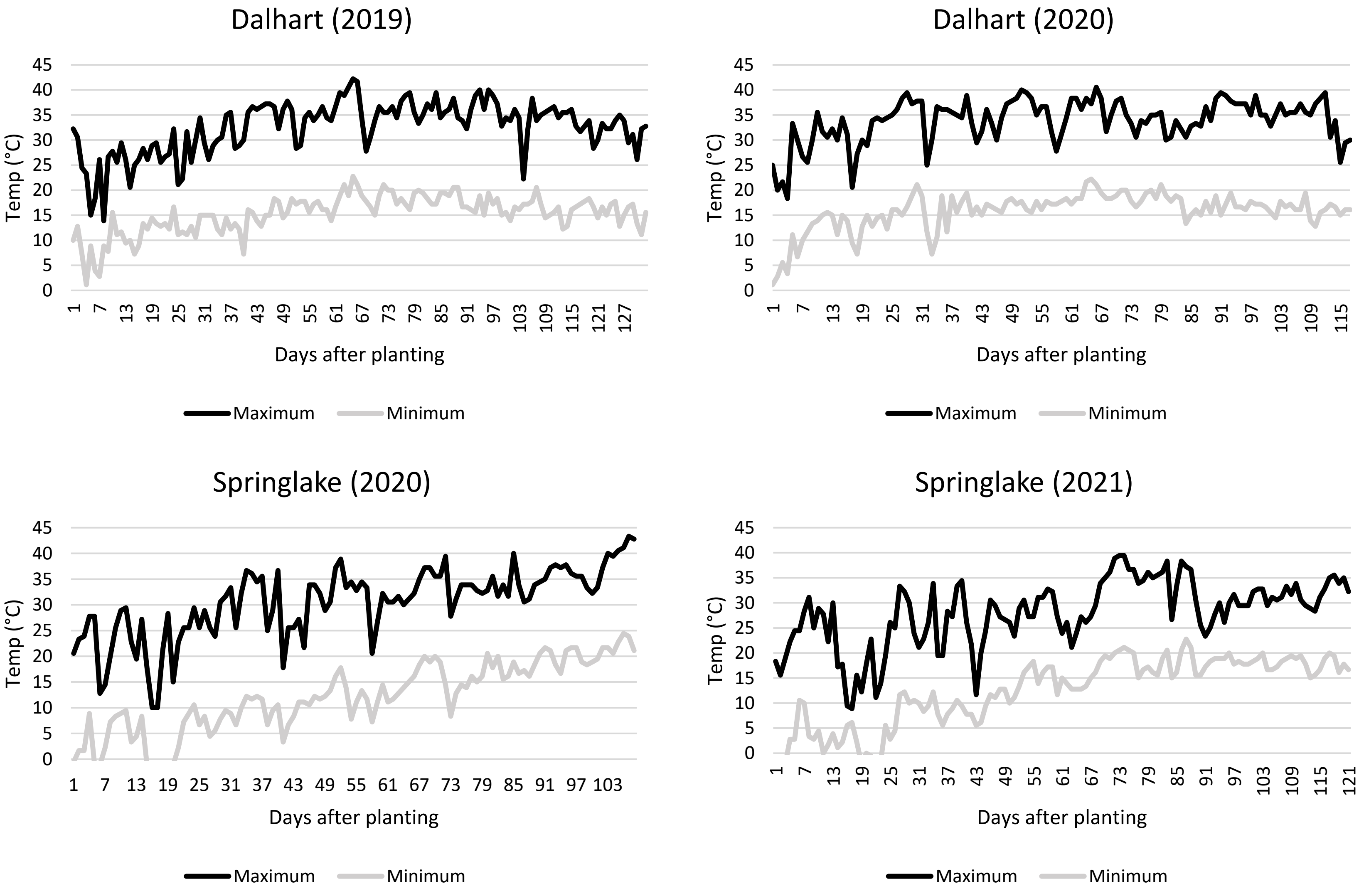

Figure S1. Temperature conditions during the potato crop period in Dalhart (2019 and 2020) and Springlake (2020 and 2021), Texas.

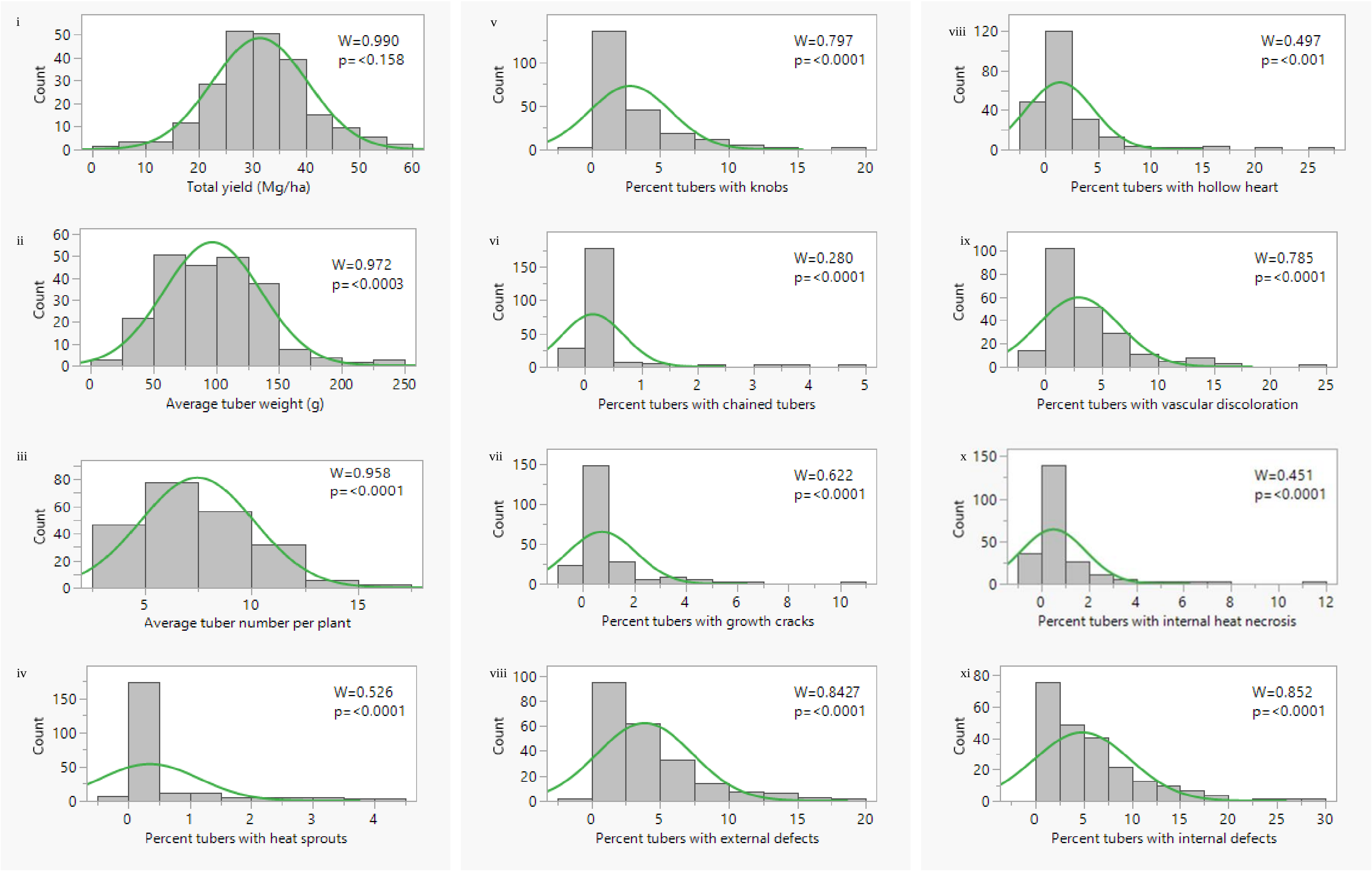

Figure S2. Distribution of potato yield and tuber defect traits measured in 217 potato genotypes in Dalhart (2019 and 2020) and Springlake (2020 and 2021).

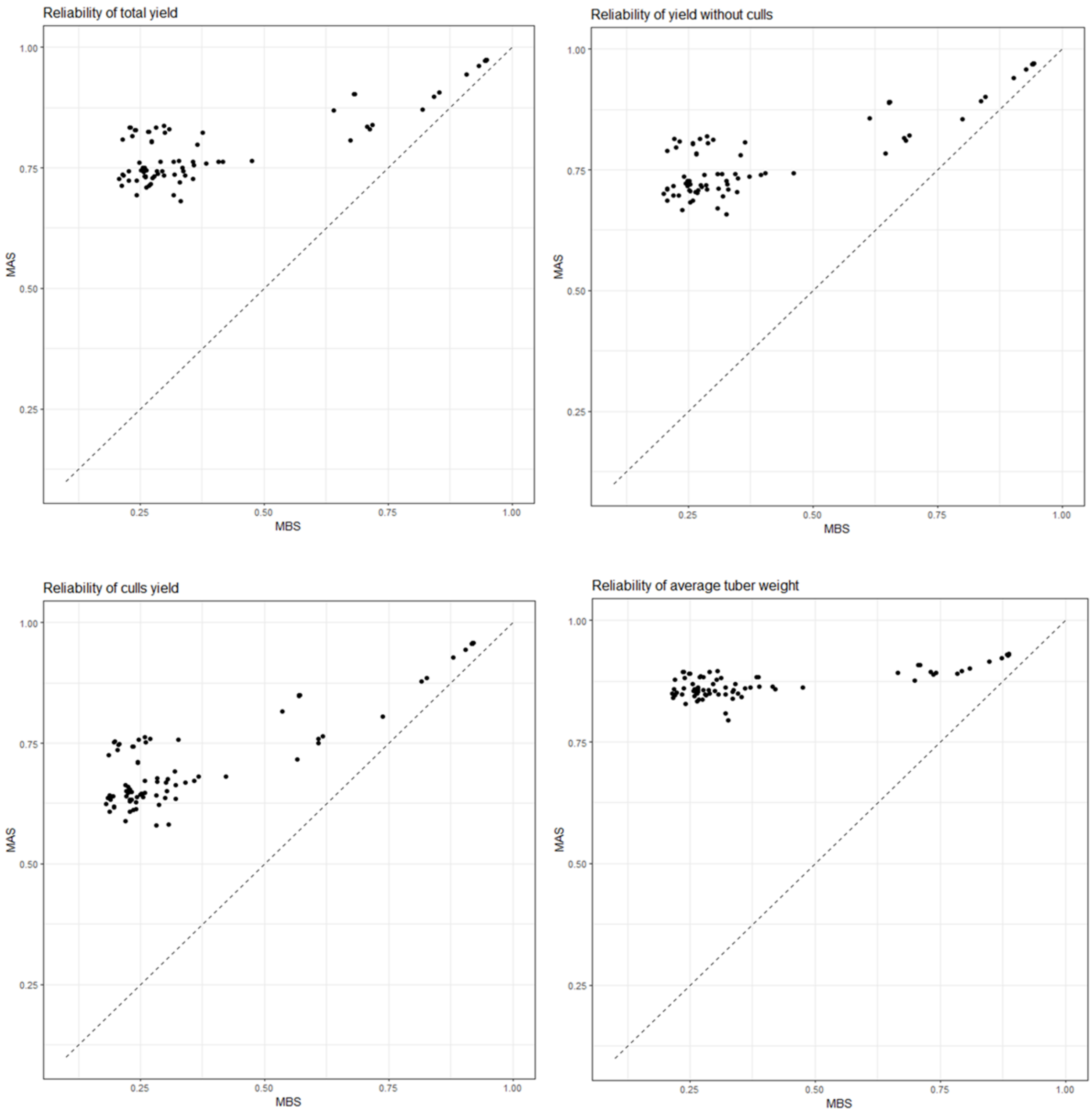
Reliability of total yield
MAS
MBS
Reliability of yield without culls
MAS
MBS
Reliability of culls yield
MAS
MBS
Reliability of average tuber weight
MAS
MBS
0.25
0.50
0.75
1.00

Reliability of tuber number per plant

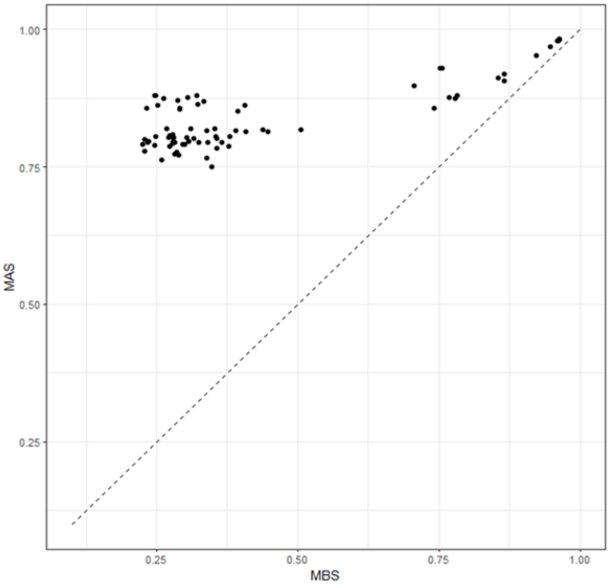

Reliability of percent tubers with heat sprouts

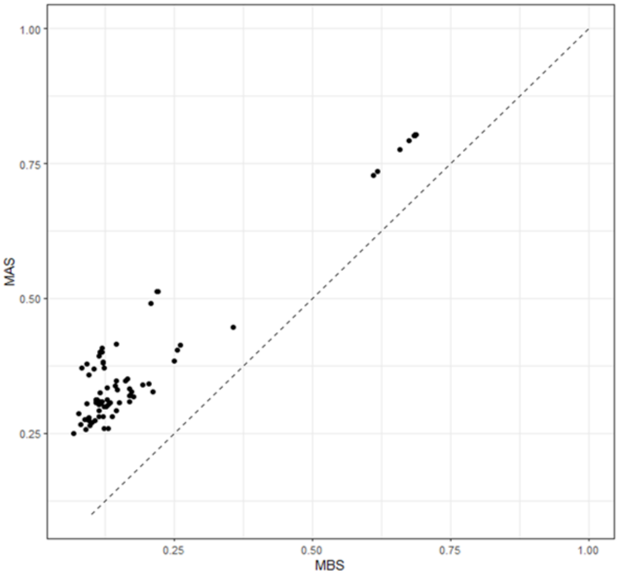

Reliability of percent tubers with knobs

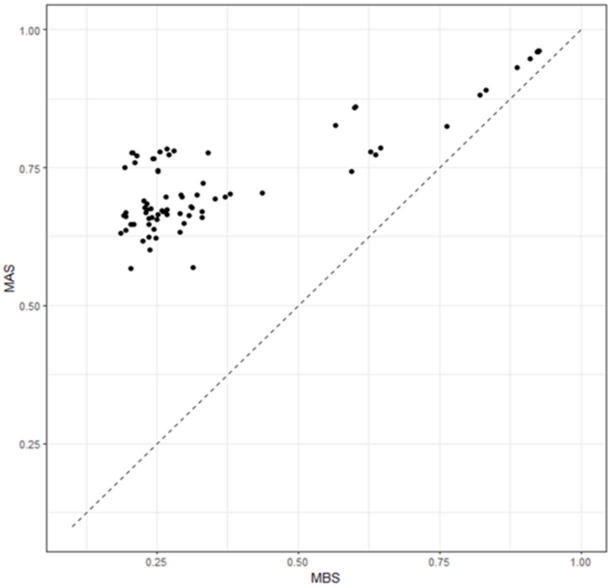

Reliability of percent tubers with chained tubers

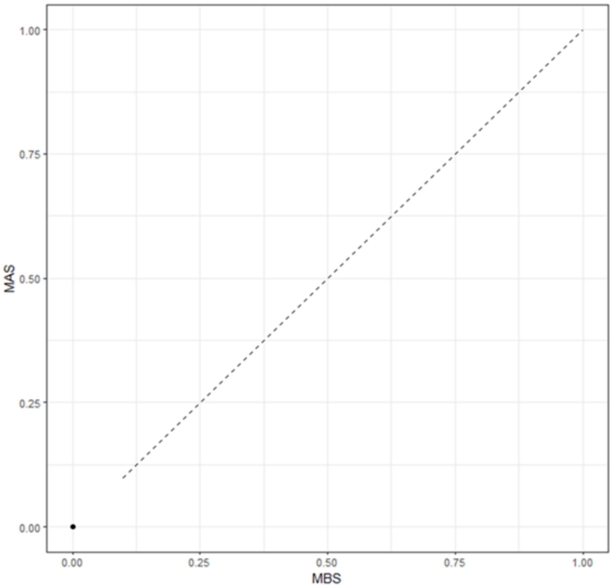

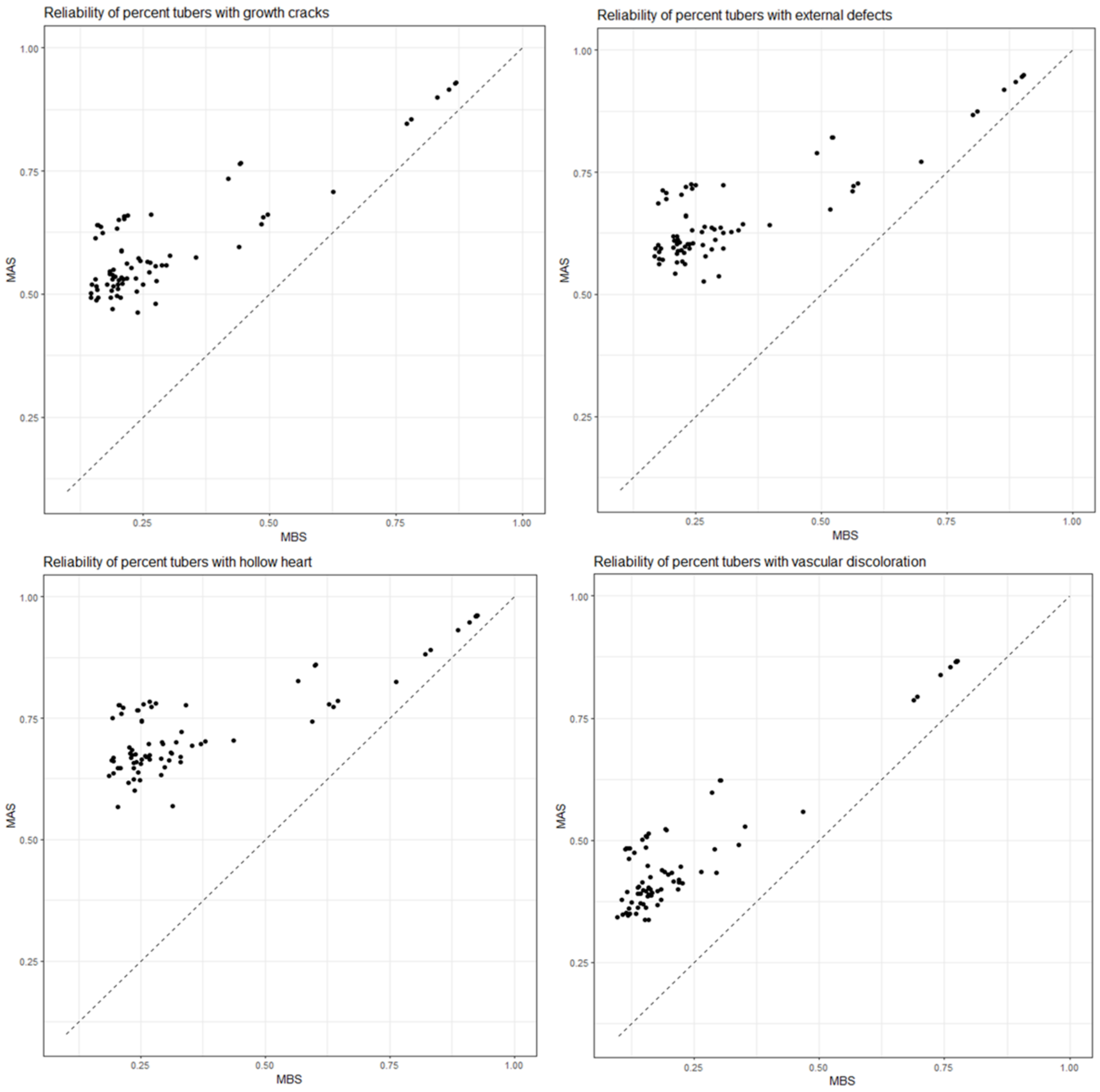
Reliability of percent tubers with growth cracks
MAS
MBS
0.25
0.50
0.75
1.00
Reliability of percent tubers with external defects
MAS
MBS
Reliability of percent tubers with hollow heart
MAS
MBS
Reliability of percent tubers with vascular discoloration
MAS
MBS

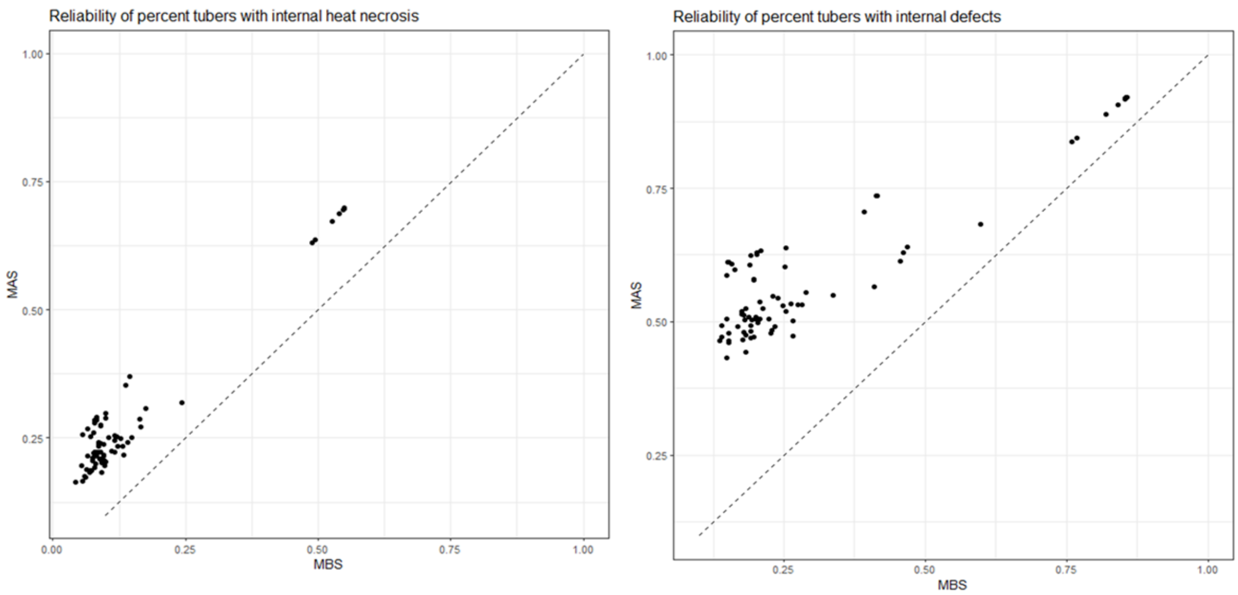

Figure S3. Comparison of the reliability of the phenotypic predictions for Aberdeen crossed clones in the dataset (86 clones) when phenotypic values were predicted based on both observed phenotype and marker data (marker-assisted selection - MAS) and when only on marker data (no observed phenotype) (marker-based selection - MBS).
